# Carbamazepine-Induced Drug Rash With Eosinophilia and Systemic Symptoms (DRESS) Syndrome

**DOI:** 10.7759/cureus.91226

**Published:** 2025-08-29

**Authors:** Nikhitha Mantri, Abeer Qasim, George S Zacharia, Sun Hoazhe, Harish Patel

**Affiliations:** 1 Gastroenterology, Jefferson Stratford Hospital, Stratford, USA; 2 Gastroenterology, BronxCare Health System, New York, USA; 3 Internal Medicine, BronxCare Health System, New York, USA; 4 Gastroenterology, Franciscan Digestive Care Associates, Tacoma, USA

**Keywords:** carbamazepine, drug hypersensitivity reaction, drug reaction with eosinophilia and systemic symptoms (dress) syndrome, eosinophilia, severe idiosyncratic drug reaction

## Abstract

Drug rash with eosinophilia and systemic symptoms (DRESS) syndrome is a severe and potentially life-threatening adverse reaction to medications. The exact underlying mechanisms of DRESS syndrome remain incompletely understood. The most frequent manifestations include fever, cutaneous rash, lymphadenopathy, and deranged hepatic biochemistry. Timely identification and cessation of the offending drug is critical in the effective management of the syndrome. Most patients with DRESS syndrome experience complete recovery upon discontinuation of the causative drug. A subset of patients may experience a protracted course with relapses and autoimmune complications, such as autoimmune thyroiditis or type 1 diabetes, necessitating long-term monitoring. Here, we report a case of carbamazepine-induced DRESS syndrome. In this case, prompt cessation of carbamazepine and treatment with systemic steroids led to complete resolution of symptoms and normalization of liver function. This case underscores the importance of promptly recognizing and managing this condition to ensure favorable outcomes and patient safety.

## Introduction

Carbamazepine is a neuropsychiatric medication widely utilized for the management of seizures, neuropathic pain, and psychiatric illnesses [1]. It acts by modulating sodium voltage-gated channels, resulting in the inhibition of action potentials and attenuation of synaptic transmissions. While carbamazepine is an efficacious drug, it is essential to be cognizant of its potential side effects, including blood dyscrasias, hepatotoxicity, cardiac dysfunction, and hypersensitivity reactions. Among the severe hypersensitivity reactions associated with carbamazepine, Stevens-Johnson syndrome, toxic epidermal necrolysis, and drug rash with eosinophilia and systemic symptoms (DRESS) syndrome are notable [1,2]. In this report, we present a case of DRESS syndrome in a middle-aged female who was initiated on carbamazepine to manage trigeminal neuralgia. DRESS syndrome is a rare drug reaction, typically characterized by the triad of fever, skin rash, and lymphadenopathy. This can be complicated by internal organ dysfunction, most commonly hematologic and hepatic, and less frequently by renal, pulmonary, cardiac, gastrointestinal, and neurological involvement [2]. Carbamazepine-induced DRESS syndrome is a rare phenomenon, with an estimated incidence of 0.7% of all reported drug reactions [3].

## Case presentation

A 57-year-old Hispanic female presented to the emergency department with a one-day history of lower abdominal pain, rash, nausea, vomiting, and diarrhea. She reported developing an erythematous, pruritic rash initially on her trunk and extremities, which later worsened and progressed to her face the next day. Concurrently, she experienced intermittent crampy lower abdominal pain associated with three to four episodes of non-bloody diarrhea. The patient also reported generalized fatigue for the past week. She was diagnosed with trigeminal neuralgia around two months before the current presentation and was initiated on carbamazepine. Her comorbidities included hypertension, hyperlipidemia, cholelithiasis, and antiphospholipid antibody syndrome complicated with recurrent lower extremity deep vein thrombosis, necessitating lifelong anticoagulation. Apart from carbamazepine, her long-term medications were warfarin, lisinopril, and atorvastatin, which she had been using for more than a decade and reported no recent change in dosing. She reported a history of allergic reaction to penicillin. She denied tobacco, alcohol, or drug use, recent travel, participation in recreational activities, or sick contacts. The family history was noncontributory.

Upon arrival, she had a low-grade fever of 100.2°F and tachycardia at 108 beats per minute. She was maintaining an oxygen saturation of 96-98% on room air and was normotensive. Diffuse erythematous non-blanching maculopapular rash was observed on the trunk, extremities, and face. She was noted to have nontender cervical and inguinal lymphadenopathy. The abdomen was nontender with no appreciable mass or organomegaly. The rest of the physical examination was unrevealing.

Hemogram at admission revealed leukocytosis and eosinophilia. The biochemical panel revealed elevated C-reactive protein (21 mg/L) and deranged hepatic function: alanine aminotransferase at 577 U/L, aspartate aminotransferase at 554 U/L, alkaline phosphatase at 405 IU/L, total bilirubin at 0.7 mg/dL, direct bilirubin at 0.6 mg/dL, and gamma-glutamyl transferase at >1,200 U/L (Table 1).

**Table 1:** A summary of the hematological and biochemical workup.

Parameter	Results				Reference range
	Baseline	Admission	Day 7	Follow-up	
Hemoglobin	11.1	10.7	10.9	10.9	12–16 g/dL
Leukocyte count	7.8	14.2	11.4	8.2	4.8–10.8 k/μL
Eosinophil count	0.4	1.8	1.1	0.5	<0.5 k/μL
Platelets	323	422	378	290	150–400 k/μL
Sodium/Potassium	139/4.5	134/4.9	136/4.5	135/4.7	135–145/3.5–5 mEq/L
Blood urea nitrogen	18	22	25	13	7–20 mg/dL
Creatinine	0.9	1.3	1.2	0.7	0.5–1.5 mg/dL
Bilirubin total/direct	0.5/0.2	0.7/0.6	0.6/0.4	0.4/0.2	0.2–11/0–0.3 mg/dL
Aspartate aminotransferase	28	544	112	32	9–36 U/L
Alanine aminotransferase	34	577	132	31	5–40 U/L
Alkaline phosphatase	76	405	175	83	43–160 U/L
Gamma-glutamyl transferase	--	>1,200	840	48	5–35 U/L
Albumin	3.9	3.4	3.4	3.7	3.5–5.5 g/dL
C-reactive protein	--	21	--	<5	<5 mg/dL

A review of the patient’s medical records revealed normal liver function test results four months before the current presentation. Abdominal ultrasound demonstrated gallstones and hepatosplenomegaly. Computed tomography and magnetic resonance cholangiopancreatography excluded additional structural hepatobiliary or pancreatic pathology. Evaluation for hepatotropic and nonhepatotropic infections was negative, as were the autoimmune markers (Table 2).

**Table 2:** An excerpt of the etiological workup. IgM: immunoglobulin M; PCR: polymerase chain reaction

Test	Results
Hepatitis A antibody (IgM)	Negative
Hepatitis B surface antigen	Negative
Hepatitis B core antibody (IgM)	Negative
Hepatitis C antibody	Negative
Hepatitis E antibody (IgM)	Negative
Epstein-Barr virus DNA PCR	Not detected
Cytomegalovirus DNA PCR	Not detected
Herpes simplex virus DNA PCR	Not detected
HIV serology	Negative
Leptospira antibody (IgM)	Negative
Human herpesvirus 6 DNA PCR	Not detected
Anti-nuclear antibodies	Negative
Anti-smooth muscle antibodies	Negative
Anti-liver-kidney-microsomal antibodies	Negative
Anti-mitochondrial antibodies	Negative`

**Figure 1:**
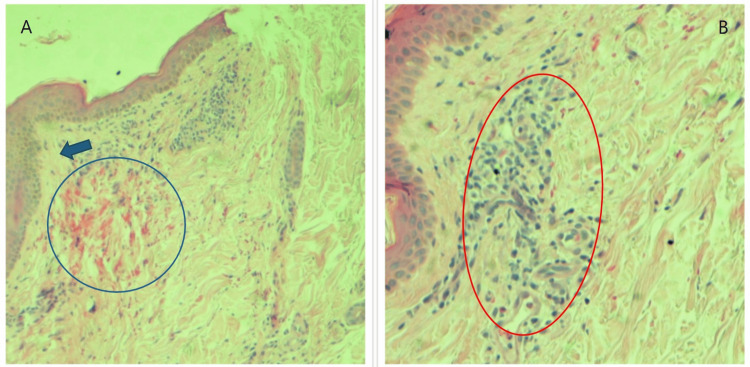
Skin biopsy microphotographs. (A) Low-power and (B) high-power images demonstrating perivascular dermatitis comprising predominantly lymphocytes and occasional eosinophils (red oval), spongiosis (blue arrow), and red blood cell extravasation (blue circle).

The possibility of a drug hypersensitivity reaction was considered, given the chronological association between the initiation of carbamazepine and the subsequent onset of rash. The RegiSCAR scoring system (Table 3) totaled 6 points, indicating a diagnosis of DRESS syndrome. A skin biopsy of the left thigh was performed on the second day of admission, which was reported two weeks later as perivascular dermatitis with hemorrhage, consistent with the diagnosis of DRESS syndrome (Figure 1).

**Table 3:** RegiSCAR criteria for the diagnosis of DRESS syndrome. RegiSCAR: European Registry of Severe Cutaneous Adverse Reaction; DRESS: drug rash with eosinophilia and systemic symptoms

Criterion	Definition/Finding	Score
Fever	Temperature ≥38.5°C	+1
Lymphadenopathy	Involvement of ≥2 sites with nodes ≥1 cm	+1
Eosinophilia	700–1,499/µL or 10–19.9%	+1
	≥1,500/µL or ≥20%	+2
Atypical lymphocytes	Present in blood film	+1
Skin rash	Rash covering ≥50% body surface area	+1
	Rash suggestive of DRESS (infiltration, facial edema, purpura)	+1
Organ involvement	1 organ (liver, kidney, lung, heart, etc.)	+1
	≥2 organs	+2
Disease course	Resolution ≥15 days	+1
Other causes excluded	Alternative diagnoses ruled out	+1
Total score: <2: not; 2–3: possible; 4–5: probable; ≥6: definite		

Carbamazepine was withheld, and she was initiated on oral prednisone, dosed at 1 mg/kg. Over the next week, the rashes, gastrointestinal symptoms, and hepatic functions improved; subsequently, she was discharged. Upon follow-up at the dermatology clinic, four weeks after discharge, it was noted that the rash had completely resolved. Additionally, there was an improvement in transaminase levels to the baseline, indicating a positive effect on liver function following treatment.

## Discussion

The DRESS syndrome is a rare, potentially life-threatening, idiosyncratic, drug hypersensitive reaction with a mortality rate of approximately 10% to 20% [2,4]. Chaiken et al. first reported a case of DRESS syndrome in 1950 involving a patient who experienced elevated transaminases, rash, and fever following the administration of phenytoin [5]. The exact mechanism of DRESS syndrome is yet to be fully elucidated; however, in cases linked to the use of anticonvulsant drugs such as phenytoin, carbamazepine, and phenobarbital, which rely on the enzyme epoxide hydrolase for drug detoxification, a possible abnormality or deficiency in this enzyme is thought to cause the accumulation of harmful toxins, leading to cellular damage and triggering adverse immunological reactions [2]. Additional proposed mechanisms include the reactivation of human herpesvirus 6, cytomegalovirus, or Epstein-Barr virus, as well as a genetic predisposition to drug hypersensitivity syndrome [6]. Specific human leukocyte antigen (HLA) haplotypes have been linked with DRESS syndrome and drugs: HLA-B*5701 (abacavir), HLA-DR3, HLA-DQ2, HLA-A*3101 (carbamazepine), and HLA-B*5801 (allopurinol) [7]. The potential triggers to DRESS syndrome include, but are not limited to, anticonvulsants, antidepressants, sulfonamides, anti-inflammatory drugs, and antibiotics. The aromatic anticonvulsants phenytoin, carbamazepine, phenobarbital, and primidone are well-known culprits that can lead to DRESS syndrome [7]. The diagnosis of DRESS should be considered in all patients with an acute cutaneous reaction accompanied by systemic manifestations, including fever, lymphadenopathy, eosinophilia, and organ dysfunction, who have a history of new drug therapy within the preceding two to eight weeks [8].

There is no single diagnostic test or gold standard for diagnosing DRESS syndrome; instead, the diagnosis of DRESS syndrome relies on diagnostic criteria. The RegiSCAR criteria are the most comprehensive and widely accepted diagnostic criteria [9]. Skin biopsy can provide valuable insights into the diagnosis of DRESS syndrome; however, the results can be nonspecific and varied. The histopathological findings may include basal vacuolization, spongiosis with focal sub-epidermal pustules, lichenoid dermatitis revealing infiltrates in the cells, perivascular infiltration, perivascular dermatitis, and even leukocytoclastic vasculitis [10]. The management of DRESS syndrome involves the immediate discontinuation of the suspected drug that triggered the adverse reaction. In case of mild disease with no or mild organ involvement (stage 1 drug-induced liver injury (DILI) or acute kidney injury), high-potency topical steroids and supportive care will suffice. In more severe DILI, an intensive level of care with systemic steroids will be required in most cases. Oral prednisone at a minimum dose of 1 mg/kg/day is effective in most cases [8]. In case of inadequate response with systemic steroids, other options include cyclophosphamide, cyclosporine, intravenous immunoglobulin, and plasmapheresis; however, the evidence is limited to case reports. There is limited data on ganciclovir or valganciclovir in severe cases wherein viral reactivation has been demonstrated or is strongly suspected [7,8,11].

Our patient presented with cutaneous lesions involving over 50% of body surface area, with concomitant lymphadenopathy and hepatic involvement following recently instated carbamazepine therapy for trigeminal neuralgia; overall consistent with the diagnosis of DRESS syndrome. The estimated RegiSCAR score was 6, suggesting a *definite diagnosis*. Furthermore, the skin biopsy was also compatible with the diagnosis of DRESS syndrome in our patient. Apart from prompt cessation of carbamazepine, she was administered oral steroids, which provided her symptomatic relief and biochemical improvement. She is planned for further follow-up in the clinic to evaluate for any possible new-onset autoimmune diseases, which could complicate DRESS syndrome.

The differential diagnosis of DRESS syndrome includes Kawasaki disease, Stevens-Johnson syndrome, toxic epidermal necrolysis, hypereosinophilic syndrome, acute generalized exanthematous pustulosis, and erythroderma. To distinguish between these conditions, various factors are considered, such as the onset of symptoms, the type of rash observed, and the extent of visceral involvement [12]. Stevens-Johnson syndrome and toxic epidermal necrolysis typically cause blistering skin lesions, skin peeling, and mucosal involvement, which was absent in our patient. Furthermore, high eosinophil counts are infrequent in patients with severe blistering skin diseases. Differentiation of Kawasaki disease from DRESS can be challenging; however, the chronological association of rash with carbamazepine intake favors a diagnosis of DRESS. Additionally, the classical features of Kawasaki disease, such as strawberry tongue and conjunctivitis, were not observed in our patient. Idiopathic hypereosinophilic syndrome is a chronic disease with insidious clinical features and rarely presents as an acute syndrome like our case. Given the complexity and potential severity of DRESS syndrome, accurate diagnosis and differentiation from similar conditions are paramount to ensure appropriate management and treatment for the patient’s condition.

## Conclusions

DRESS syndrome is a potentially life-threatening drug hypersensitivity reaction, frequently associated with aromatic anticonvulsants, including carbamazepine. Healthcare providers should maintain a high level of clinical suspicion for DRESS syndrome in patients with new-onset cutaneous eruptions, with or without systemic manifestations, that develop within weeks of introducing a new drug. Prompt cessation of the implicated drug combined with systemic steroids forms the backbone of management in DRESS syndrome.
